# Supplementary material for: Content-rich biological network constructed by mining PubMed abstracts
Source: BMC Bioinformatics. 2004 Oct 8;5:147. doi: 10.1186/1471-2105-5-147 (PMC528731; doi:10.1186/1471-2105-5-147)
Supplement: Additional File 2 — The original results of the above study (non-essential files are deleted to keep the file size under the limit set by BMC bioinformatics). [file 1471-2105-5-147-S2.bz2 › chilibotAdditionalFile2/dip05/614ID10619841E10997/html/GTF2B_TBP.html]

 


 **GTF2B** and **TBP** 
  
Found 354 abstracts in PubMed, retrieved 05.  
 

 What does Google say? 
 PDF only 
| .edu only 

---

**Interactive relationship** (e.g. stimulation, inhibition, etc)

**Inhibitory relationship**- We also found through chromatin immunoprecipitation experiments that the interaction between NZFP and  **TBP**  inhibits binding of TFIIA and TFIIB  [ **GTF2B** ] .  Ref: 12788093 Biochem Biophys Res Commun, 2003
- A novel  **TBP**  interacting zinc finger protein represses transcription by inhibiting the recruitment of TFIIA and TFIIB  [ **GTF2B** ] .  Ref: 12788093 Biochem Biophys Res Commun, 2003
**Neutral relationship**- The interacting region identified in IE62 overlaps with or borders on sites involved in interactions with the VZV IE4 protein and the cellular factors  **TBP**  and TFIIB  [ **GTF2B** ] .  Ref: 12855699 J Biol Chem< MedlineTA>J Biol Chem, 2003
- Our simulated TATA  **TBP**  complexes develop sequence dependent structure and motion trends that may lead to favorable orientations for high activity variants with respect to binding TFIIA, TFIIB  [ **GTF2B** ] , and other transcription factors, while conversely, accelerate dissociation of low activity TATA  **TBP**  complexes.  Ref: 12767124 Biopolymers, 2003
- These data strongly suggest that the repression by NZFP occurs through its binding to both DNA and  **TBP**  and the resulting NZFP  **TBP**  promoter complex inhibits preinitiation complex assembly by preventing binding of TFIIA and TFIIB  [ **GTF2B** ] .  Ref: 12788093 Biochem Biophys Res Commun, 2003

**Non-interactive relationship** (e.g. studied together, co-existance, homology, etc.)

- Native human TATA binding protein  [ **TBP** ]  simultaneously binds and bends promoter DNA without a slow isomerization step or TFIIB  [ **GTF2B** ]  requirement.  Ref: 12791683 J Biol Chem< MedlineTA>J Biol Chem, 2003
- The inhibitory effect of E1A 13S on the assembly of the PIC is overcome when excess  **TBP**  is added back in the reaction, while addition of either excess TFIIA or TFIIB  [ **GTF2B** ]  were ineffective.  Ref: 12818428 Biochim Biophys Acta, 2003
- In addition, EMSA studies show that adenovirus E1A 13S oncoprotein is unable to disrupt either the preestablished TFIIA  **TBP**  TATA or TFIIA TFIIB  [ **GTF2B** ]   **TBP**  TATA complexes, but does inhibit complex formation when all transcription factors were simultaneously added.  Ref: 12818428 Biochim Biophys Acta, 2003
